# Ascites from ovarian cancer patients stimulates MUC16 mucin expression and secretion in human peritoneal mesothelial cells through an Akt-dependent pathway

**DOI:** 10.1186/s12885-019-5611-7

**Published:** 2019-04-30

**Authors:** Isabelle Matte, Perrine Garde-Granger, Paul Bessette, Alain Piché

**Affiliations:** 1 Département de Microbiologie et Infectiologie, Université de Sherbrooke, 3001, 12ième Avenue Nord, Sherbrooke, Québec J1H 5N4 Canada; 2 Département de Pathologie, Faculté de Médecine, Université de Sherbrooke, 3001, 12ième Avenue Nord, Sherbrooke, J1H 5N4 Canada; 3 Département de Chirurgie, service de gynécologie-obstétrique, Faculté de Médecine, Université de Sherbrooke, 3001, 12ième Avenue Nord, Sherbrooke, J1H 5N4 Canada

**Keywords:** Ascites, Ovarian cancer, MUC16, Mesothelial cells

## Abstract

**Background:**

CA125 is a well-established ovarian cancer (OC) serum biomarker. The CA125 heavily glycosylated epitope is carried by the MUC16 mucin, a high molecular weight transmembrane mucin. Upon proteolytic cleavage, the extracellular domain of MUC16 is released from the cell surface into malignant ascites and blood vessels. Previous studies have shown that both tumor and surrounding mesothelial cells may express MUC16. Although little is known about the regulation of MUC16 expression in these cells, recent evidence suggest that inflammatory cytokines may stimulate MUC16 expression. Because malignant ascites is a pro-inflammatory environment, we investigated whether OC ascites stimulate the expression and release of MUC16 by human peritoneal mesothelial cells (HPMCs).

**Methods:**

HPMCs were isolated from peritoneal lavages of women operated for conditions other than cancer. MUC16 protein expression was determined by immunoblot, immunofluorescence or immunohistochemistry depending on the experiments. The release of MUC16 from the cell surface was measured using EIA and MUC16 mRNA expression by ddPCR.

**Results:**

We show that high-grade serous ascites from patients with OC (*n* = 5) enhance MUC16 expression in HPMCs. Malignant ascites, but not benign peritoneal fluids, stimulate the release of MUC16 in HPMCs in a dose-dependent manner, which is abrogated by heat inactivation. Moreover, we establish that ascites-induced MUC16 expression occurs at the post-transcriptional level and demonstrate that ascites-induced MUC16 expression is mediated, at least partially, through an Akt-dependent pathway. A cytokine array identified upregulation of several cytokines and chemokines in ascites that mediate MUC16 upregulation versus those that do not, including CCL7, CCL8, CCL16, CCL20, CXCL1, IL-6, IL-10, HGF and IL-1 R4. However, when individually tested, none of these factors affected MUC16 expression or secretion. Concentrations of CA125 in the serum of a given patient did not correlate with the ability of its corresponding ascites to stimulate MUC16 release in HPMCs.

**Conclusions:**

Collectively, these data indicate that mesothelial cells are an important source of MUC16 in the context of ovarian cancer and malignant ascites is a strong modulator of MUC16 expression in HPMCs and uncover the Akt pathway as a driving factor for upregulation of MUC16. Factors in ascites associated with enhanced MUC16 expression and release remains to be identified.

## Background

The tumor-associated antigen CA125, encoded by the *MUC16* gene, is detectable in the sera of most women with high-grade serous ovarian carcinomas (HGSOC) [1]. CA125 is an epitope located on a repeated extracellular domain of MUC16 protein [2–5]. Rising and falling levels of serum CA125 correlate with HGSOC progression and regression, making CA125 the most important clinical biomarker for this disease [6–8]. The MUC16 extracellular central domain contains > 60 glycosylated tandem repeated sequences (156 amino acid). MUC16 C-terminal domain (CTD) contains a unique extracellular region, a transmembrane domain as well as a short 31 amino acid cytoplasmic tail (CT) [2–5]. The ectodomain of MUC16 appeared to be released by metalloproteases (MMPs) and neutrophil elastases (NE) [9, 10]. However, the involvement of these enzymes in MUC16 cell surface cleavage is controversial [11].

Mucins normally function to protect and lubricate the epithelium but alterations of MUC16 expression or glycosylation have been associated with the development and progression of ovarian carcinoma [12–14]. Specifically, we showed that MUC16 knockdown in ovarian cancer cells significantly decreased tumorigenicity, whereas the enforced expression of MUC16 C-terminal domain (last 284 C-terminal residues) enhanced soft agar colony formation and tumor growth in nude mice [13]. Others have confirmed these data by showing that MUC16 knockdown in overexpressing breast or pancreatic cancer cell lines decreased cell proliferation and in vivo tumor growth [15–18]. MUC16 expression can also protect cells from the action of cytotoxic drugs [19, 20]. Furthermore, expression of MUC16 C-terminal domain induces oncogenic transformation of NIH3T3 cells [14]. The ectodomain of MUC16 may have an immunoprotective effect through its interaction with NK cell inhibitory receptor Siglec-9. Binding to Siglec-9 inhibits the interaction between NK cells and cancer cells required for NK-induced cytolysis [21].

Although MUC16 is an oncogene that plays an important role in the development and progression of ovarian cancer, the regulation of MUC16 expression is not well characterized. The expression of MUC16 is not restricted to tumor cells. It is also expressed by the mesothelial cells lining of the adult pleura, pericardium, and peritoneum [22, 23]. Human peritoneal mesothelial cells (HPMCs) have been reported to be the major source of MUC16 found in the sera of ovarian cancer patients [24]. Secretion of MUC16 in the supernatant of HPMCs was found to be about 5-fold that of ovarian cancer cell lines [24]. The concentration of MUC16 in peritoneal dialysis effluent has been used for many years as a biomarker for mesothelial cell mass in patients on peritoneal dialysis, which suggest that MUC16 expression is associated with areas of inflammation [25]. Furthermore, MUC16 expression is often increased in non-malignant inflammatory conditions [26–29]. Indeed, cytokines such IL-1β, IL-6, IL-8, IL-17, TNFα and IFNγ have been shown to alter the expression of MUC16. However, the regulation of MUC16 expression by inflammatory cytokines may differ between HPMCs and tumor cells. For example, IL-1β or TNFα treatment of HPMCs resulted in a significant reduction of MUC16 release whereas IFNγ did not influence the shedding of MUC16 in HPMCs [24]. In contrast, TNFα and IFNγ stimulated MUC16 mRNA levels in tumor cells, a process that was, at least partly, NF-κB dependent [26].

Because ovarian cancer ascites is an inflammatory environment that contains a variety of cytokines, chemokines and growth factors [30–32], we hypothesized that ascites could stimulate the expression of MUC16 and its release by HPMCs. The goal of this study was therefore to assess the effect of ascites on MUC16 expression in HPMCs. Given the role of MUC16 in ovarian cancer progression, identifying factors that regulates its expression may provide new avenues for ovarian cancer treatment.

## Methods

### Cell culture, clinical samples and reagents

Ascites was routinely obtained at the time of the debulking surgery of ovarian cancer patients treated at the Centre Hospitalier Universitaire de Sherbrooke. Peritoneal fluids were centrifuged at 1000 rpm for 15 min and cell-free supernatants were stored at − 80 °C until assayed. All acellular fluids were supplied by the Banque de tissus et de données of the Réseau de Recherche en Cancer of the Fonds de la Recherche du Québec en Santé affiliated to the Canadian Tumor Repository Network (CTRNet). This study was approved by the Institutional Review Board of the Centre de Recherche du CHUS. Informed written consent was obtained from women that underwent surgery by the gynecologic oncology service between 2000 and 2017. All samples were reviewed by an experienced pathologist. Baseline characteristics and serum CA125 levels were collected for all patients. Patients (range 36–76 years) were staged according to the criteria of the International Federation of Gynecology and Obstetrics (FIGO). The ascites characteristics are shown in Table 1. Peritoneal fluids OV401 and OV437 were obtained from benign gynecological conditions including a fibroma and a serous cystadenoma respectively.

**Table 1:** Ascites characteristics

Ascites	Sub-type	Stage	Grade	Optimal reduction	Chemotherapy before debulking surgery	MUC16 levels in ascites at baseline (KIU/ml)
OVC346	Serous	3C	3	Yes	No	7146
OVC439	Serous	3C	3	Yes	No	3453
OVC508	Serous	4	3	Yes	No	100,000
OVC509	Serous	4	2	Yes	No	1409
OVC690	Serous	2B	3	Yes	No	4463

HPMCs were isolated from peritoneal lavages of women operated for conditions other than cancer. HPMCs were grown in DMEM/F12 supplemented with 0.4 μg/ml of hydrocortisone (Sigma, Oakville, Canada), 10% EGF (Sigma, Oakville, Canada), and 10% FBS and antibiotics (penicillin and streptomycin). The media was changed every 3 days while cells were maintained at 37 °C in a humidified 5% CO_2_ incubator. The nature of HPMCs was confirmed by immunostaining with antibodies against calretinin (Life Technologies, Burlington, ON), N-cadherin, vimentin, smooth muscle actin (Santa Cruz Biotechnology Inc., Santa Cruz, CA), E-cadherin (BD Biosciences). HPMCs stained positive for mesenchymal markers such as vimentin, smooth muscle actin and for calretinin, a specific mesothelial marker. HPMCs were negative for the epithelial markers MOC31 and E-cadherin (data not shown). The OVCAR3 human ovarian cancer cell line was obtained in 2002 from the American Type Culture Collection (Manassas, VA). OVCAR3 cells were grown in RPMI 1640 (Wisent) supplemented with 20% heat-inactivated FBS (Wisent), 2 mM L-glutamine (Wisent), 100 units/ml penicillin, 100 μg/ml streptomycin and 10 μg/ml insulin, and maintained at 37 °C in a humidified 5% CO_2_ incubator. Cells were tested regularly for mycoplasma contamination.

### Immunofluorescence and immunohistochemistry

OVCAR3 cell line and HPMCs were grown on glass slides. The cells were washed 3 times before a 24 h treatment with ascites, benign fluid or TNFα (Cell Signaling Technology, Danvers, MA) in serum- and hormone-free DMEM/F12 supplemented with antibiotics and glutamine. Glass slides were then washed in cold PBS and fixed in 3.7% formaldehyde 20 min at 4 °C. Glass slides were next rinsed 5 min in cold PBS, permeabilized in PBS containing 0.1% Triton X-100 for 10 min and rinsed again in PBS. Slides were blocked in PBS/5% Goat serum 15 min and incubated with primary antibodies in staining buffer (0.02% Tween 20, 1% BSA, 1% goat serum, 5 mM EDTA, pH 7,4) at room temperature for 1 h. Slides were washed 3 times in cold PBS, incubated for 1 h at 4 °C with Alexa Fluor 594 or Alexa Fluor 488 F (ab’)2-Goat anti-Mouse IgG (H + L) or F (ab’)2-Goat anti-Rabbit IgG (H + L) (Invitrogen, Burlington, ON). Slides were then washed twice, nucleus were stained with DAPI and visualized with a Leica DM RXA fluorescence upright microscope (Leica, Wetzlar, Germany) and images were processed with Meta Imaging 7.7 software from MetaMorph. For the immunohistochemistry experiment, we used the EnVision+ System-HRP (DAB) kit from DAKO (Burlington, ON) and the protocol was performed according to the manufacturer instructions. Primary antibody anti-calretinin was from Invitrogen, anti-MUC16 and anti-alpha-Smooth muscle actin were from Dako, anti-phospho NF-kB p65 and anti-NF-kB p65 were from Cell Signaling Technology.

### CA125 measurements

CA125 was determined at Centre Hospitalier Universitaire de Sherbrooke laboratory in cell-free culture supernatant samples by EIA using the Roche Modular E170 analyzer and CA125 II regents (Roche Diagnostics, Laval, QC). Values are expressed in kilo Unité Internationale/Liter (kUI/L).

### MUC16 transcript levels

Droplet Digital PCR (ddPCR) reactions for MUC16 are composed of 10 μl of 2X QX200 ddPCR EvaGreen Supermix (Bio-Rad, Hercules, CA), 10 ng (3 μl) cDNA, 100 nM final (2 μl) primer pair solutions and 5 μl molecular grade sterile water (Wisent) for a 20 μl total reaction. ddPCR fourplex reactions for reference genes are composed of 10 μl of 2X QX200 ddPCR Supermix for probe (Bio-Rad), 10 ng (3 μl) cDNA, 250 nM final probe solutions for MRPL19 (FAM, IDT, Coralville, IA) and YWHAZ (HEX, IDT), 125 nM final probe solutions for PUM1 (FAM, IDT) and B2M (HEX, IDT), 0.9 μM final primer pair solutions for each reference genes for a 20 μl total reaction. Each reaction mix (20 μl) was converted to droplets with the QX200 droplet generator (Bio-Rad). Droplet-partitioned samples were then transferred to a 96-well plate, sealed and cycled in a C1000 deep well Thermocycler (Bio-Rad) under the following cycling protocol: 95 °C for 5 min (DNA polymerase activation), followed by 50 cycles of 95 °C for 30 s (denaturation), 59 °C for 1 min (annealing) and 72 °C for 30 s (extension) followed by post-cycling steps of 4 °C for 5 min and 90 °C for 5 min (Signal stabilization)) and an infinite 12 °C hold for MUC16 reactions. Reference gene reactions were cycled under the following cycling protocol: 95 °C for 5 min (DNA polymerase activation), followed by 50 cycles of 94 °C for 30 s (denaturation), 59 °C for 1 min (annealing/extension) followed by post-cycling steps of 98 °C for 10 min (enzyme deactivation) and 4 °C for infinite (Hold). The cycled plate was then transferred and read using the QX200 reader (Bio-Rad) either the same or the following day post-cycling. The concentration reported is copies/μl of the final 1x ddPCR reaction (using QuantaSoft software from Bio-Rad) [33].

### Conditioned media

To generate HPMCs-conditioned media, HPMCs were cultured in twelve-well plates in complete medium until they reached 80% density. Cultured media was removed, cells were washed twice, starved for 4 h in growth hormone and FBS-free medium and treated overnight in growth hormone free medium containing no FBS, FBS 10%, benign fluid 10%, malignant ascites 0.001–10%, malignant ascites 10% heat inactivated at 100 °C for 10 min, TNF-alpha (20 ng/ml) (New England Biolabs, Whitby, ON), IL-10 (1 ng/ml), IL-6 (2 ng/ml), HGF (1 ng/ml), CCL18 (20 ng/ml), CCL7, CCL8, CCL16, CCL20, CXCL1, IL1-R4 (all at 10 ng/ml) (Peprotech, Rocky Hill NJ), Leptin (0.5 ng/ml) (RnD Systems, Minneapolis MN), Actinomycin D (8 nM), NF-kB Inhibitor BAY117082 (Sigma), anti-β1 Integrin or anti-αvβ5 (5 μg/ml) (EMD Millipore, Burlington, MA). When inhibitors or actinomycin D were used, cells were preincubated 1 h prior the treatment with the inhibitor alone and then treated overnight as described. Cells were washed three times and fresh medium without FBS nor growth factors were added in each well. HPMCs were cultured for 24–96 h and medium conditioned by stimulated HPMCs were subjected to CA125 quantification. Results were normalized to the total protein concentration in the cell lysate and expressed as kilounits of MUC16 per gram of total proteins.

### Western blot analysis

HPMCs were rinsed twice in PBS, starved for 4 h in serum and growth hormone free medium and treated overnight in growth hormone free medium containing no FBS, FBS 10%, benign fluid 10%, malignant ascites 10%, TNF-alpha (20 ng/ml) (New England Biolabs), Actinomycin D (8 nM), NF-kB Inhibitor BAY117082 (Sigma) or AKT Inhibitor 1 L-6-hydroxymethyl-*chiro*-inositol2(R)-2-*O*-methyl-3-*O*-octadecylcarbonate (Sigma). When inhibitors or actinomycin D were used, cells were preincubated 1 h prior the treatment with the inhibitor alone and then treated overnight as described. Cells were washed with ice-cold PBS and whole cell extracts were prepared in lysing buffer (Glycerol 10%, Triton X-100 1%, TRIS 10 mM pH 7.4, NaCl 100 mM, EGTA 1 mM, EDTA 1 mM, SDS 0.1%) containing protease inhibitors (0.1 mM AEBSF, 5 μg/ml pepstatin, 0.5 μg/ml leupeptin and 2 μg/ml aprotinin) and phosphatase inhibitors (Na_4_P_2_O_7_ 20 mM, NaF 1 mM, Na_3_VO_4_ 2 mM). Proteins were separated by 10% SDS-PAGE gels. Proteins were transferred to PVDF membranes (Roche, Laval, QC) by electroblotting, and immunoblot analysis was performed as previously described (13). All primary antibodies were incubated overnight at 4 °C in 5% fat-free milk. Proteins were visualized by enhanced chemiluminescence (GE Healthcare, Baie d’Urfé, QC). Proteins quantification was performed using ImageJ software by National Institutes of Health. Anti-phospho-Akt (Ser483) (#44-621G), anti-pFAK and anti-pY418 Src antibodies were purchased from Life Technologies (Burlington, ON, Canada). HRP-conjugated anti-mouse (#7076) and –rabbit (#7074) antibodies, and Akt (#9272) antibodies were from Cell Signaling Technology. Anti-CA125 M11 antibody was obtained from Dako, anti-GAPDH and anti-Tubulin were from Sigma. Anti-Src was from Thermo Fisher scientific (Burlington, ON, Canada).

### Ascites analysis using multiplex cytokine array

Cytokine levels in peritoneal fluid samples were determined using human cytokine antibody assay (G series 1000) from RayBiotech Inc. (Norcross, GA). This multiplex assay measures simultaneously 120 different cytokines on a glass chip format. In this method, the levels of cytokines are expressed as relative fluorescent units and can be used to compare cytokine levels in different ascites. This method does not provide the concentration (pg/ml) of cytokines. The signal intensities were quantified using the ScanArray express dual-color confocal laser scanner (Perkin Elmer). Data were collected in Cy3 channel and stored as paired TiFF images. Spots were identified and local background substracted using the TiGR_Spotfinder 3.1.1 software. By comparing the signal intensities, relative levels of cytokines can be established.

### Statistical analysis

Comparison between unpaired groups was possible using the Mann-Whitney test or the Kruskal-Wallis test. The threshold for statistical significance is *P* <  0.05.

## Results

### Basal MUC16 expression and secretion in HPMC monolayer cultures

**Fig. 1:**
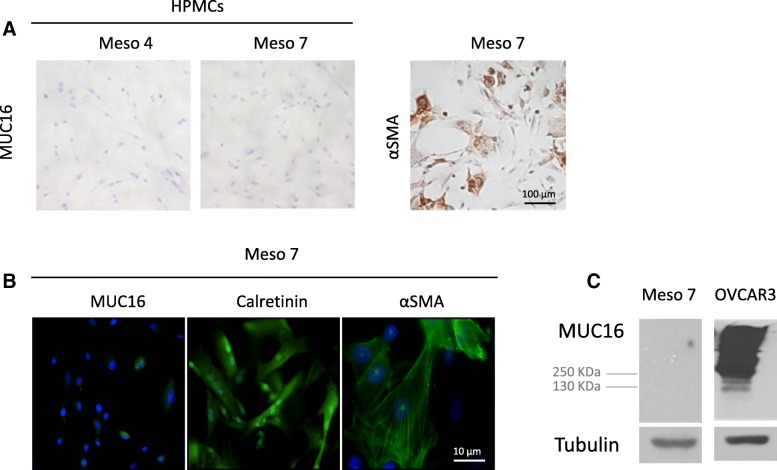
Expression of MUC16 in HPMCs. **a** Immunohistochemistry staining for MUC16 on two different HPMCs samples (Meso 4 and Meso 7) grown on glass slides using anti-CA125 M11 antibody (left panel). HPMC Meso 7 were cultured on a glass slide and stained with anti-αSMA antibody (right panel). Representative images are shown. **b** Immunofluorescence detection of MUC16, calretinin and αSMA in Meso7 HPMCs. The cells were fixed, permeabilized and incubated with anti-CA125 M11, anti-calretinin or anti-αSMA antibodies. HPMCs stained positive for calretinin and αSMA but negative for MUC16 confirming their mesenchymal phenotype. Representative images are shown. **c** Expression of MUC16 in Meso7 HPMCs and ovarian cancer cell line OVCAR3 was determined by immunoblot using anti-CA125 M11 antibody. Tubulin was used as a loading control. The ovarian cancer cell line OVCAR3 was used as a positive control for MUC16 expression

Constitutive synthesis and shedding of MUC16 glycoprotein have been previously reported in HPMC monolayer cultures suggesting that HPMCs may be an important source of the MUC16 found in the ascites and sera of women with ovarian cancer [24]. We thus determine the basal MUC16 protein expression in primary HPMC monolayer cultures (*n* = 2). Primary HPMCs did not constitutively expressed MUC16 as demonstrated by immunohistochemistry staining (Fig. 1a). However, HPMCs stained strongly for α smooth muscle actin (αSMA), a myofibroblastic marker, and calretinin, a specific mesothelial marker (Fig. 1a and b). In line with these data, MUC16 expression was not detected by immunofluorescence microscopy (Fig. 1b) or by immunoblot analysis in HPMC cultures (Fig. 1c). The release of MUC16 in HPMCs cultures was barely detectable in the supernatant of HPMC cultures (range 1 to 1.2 kUI/L). These data demonstrate that HPMCs do not show constitutive MUC16 expression and release in primary HPMC monolayer cultures.

### Ascites stimulate MUC16 protein expression and secretion in HPMCs

**Fig. 2:**
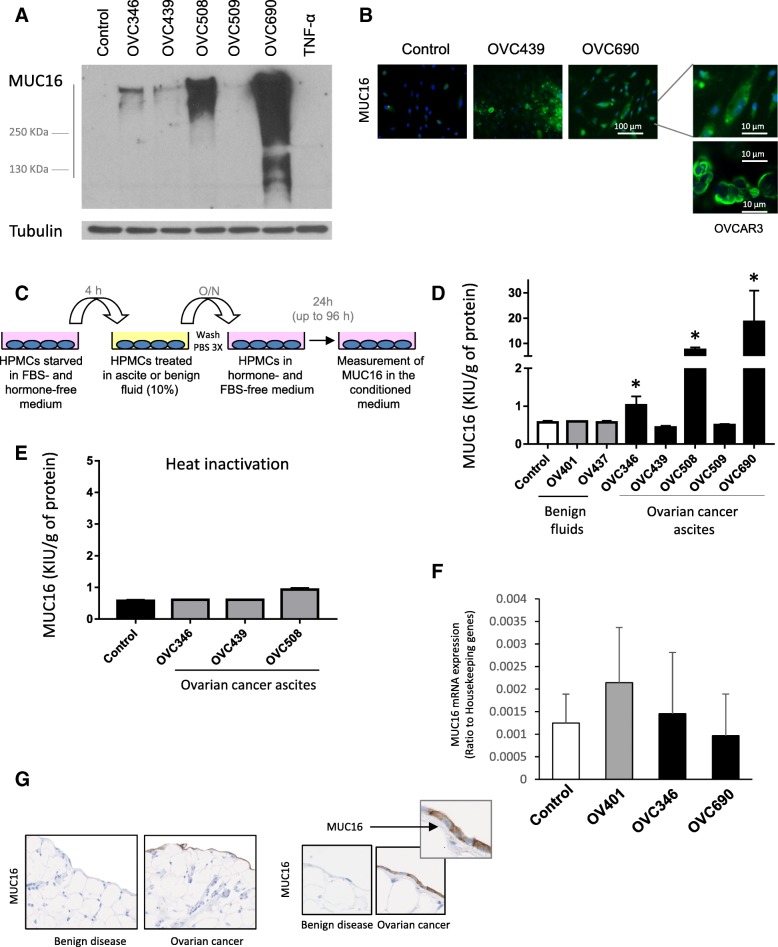
Ovarian cancer ascites stimulates MUC16 expression and release in HPMCs. **a** Meso7 HPMCs were cultured for 24 h in the presence of different ascites (10% *v*/v), TNFα (25 ng/ml) or FBS 10% (control). Cell lysates were obtained and immunoblot were performed to assess MUC16 expression. Tubulin was used as a loading control. **b** Immunofluorescence detection of MUC16 in Meso7 HPMCs. Meso7 cells were primed with OVC439 and OVC690 ascites (10% v/v) for 24 h and cells were fixed with 3.7% formaldahyde, permeabilized with 0.1% triton and stained with anti-CA125 M11 antibody (left panel). OVCAR3 cells were treated similarly. Magnification of Meso7 treated with OVC690 ascites and OVCAR3 cells stained with anti-CA125 M11 antibody (right panel). **c** Schematic representation of HPMCs experimental approach for the measurement of MUC16 in conditioned medium. HPMCs were incubated in medium containing 10% FBS until confluence was reached. Cells were then washed, starved for 4 h, treated with different ascites or benign fluids as indicated and incubated overnight. Cells were then washed 3 times and incubated in serum-free medium for an additional 24 h or more. The conditioned medium was collected, centrifuged and the supernatants were assessed for MUC16 detection. **d** Levels of MUC16 in the supernatant of conditioned medium of treated Meso7. Levels were standardized according to the total protein concentration. Results are from two independent experiments performed in duplicates. * indicates *P* <  0.001. **e** Ascites were heat-inactivated by heating at 100 °C for 10 min followed by centrifugation at 13,000 rpm for 15 min. HPMCs were incubated with inactivated ascites or medium containing 10% FBS (control). Results are from two independent experiments performed in duplicates. **f** Meso7 cells were starved in medium without FBS nor hormones for 4 h and then incubated 4 or 8 h with medium containing either 10% FBS (control), 10% benign fluid OV401 or 10% ascites (OVC346, OVC690). MUC16 mRNA levels were quantified by ddPCR. MUC16 concentration data from ddPCR experiments are expressed as ratio against reference genes for each sample. *P* > 0.05. Results are from a single experiment performed in triplicates. **g** Immunohistochemistry of MUC16 on human omentum section, in benign disease versus ovarian cancer conditions (Picture taken at 10X magnification, left panel). The counterstaining was done with haematoxylin to allow nuclei visualization. Representative images from omental biopsies from two ovarian cancer and one patient with a benign disease were obtained and multiple section were analyzed. Enlarged representative images (40X magnification; right panel) shows the localisation of MUC16 at the mesothelium in ovarian cancer omentum

HGSOC ascites are a pro-inflammatory tumor environment that contains a variety of cytokines, chemokines and growth factors including leptin, hepatocyte growth factor (HGF), IL-6, and CCL18 [30–32, 34]. Through binding to specific cell surface receptors, these factors may activate changes in the expression pattern of molecules essential for ovarian cancer progression. Because pro-inflammatory cytokines have been previously shown to stimulate expression of membrane-bound mucins such as MUC1, MUC4, and MUC16 in tumor cells [26–29, 35–37], we hypothesized that ascites could enhance MUC16 expression in HPMCs. We thus determined the effect of different HGSOC ascites (*n* = 5) on MUC16 expression and secretion. The characteristics of these ascites are shown in Table 1. In the absence of ascites, little or no MUC16 expression was detectable by immunoblot (Fig. 2a). In contrast, HGSOC ascites stimulated the expression of MUC16 protein in HPMCs, although the degree of stimulation markedly varied between ascites. TNFα (25 ng/ml), which has been previously reported to stimulate MUC16 expression in breast and ovarian cancer cells [26], failed to increase MUC16 protein expression in HPMCs (Fig. 2a). Immunofluorescence analysis also demonstrated the stimulation of MUC16 expression by OVC439 and OVC690 ascites in HPMCs (Fig. 2b). In MUC16 overexpressing epithelial ovarian cancer cell lines such as OVCAR3, MUC16 expression distribution is mainly limited to the cell surface (Fig. 2b). In ascites-stimulated HPMCs, MUC16 localization was more diffuse (Fig. 2b). We next measured the release of MUC16 in the conditioned medium of ascites-primed HPMCs (Fig. 2c). There was a robust stimulation of MUC16 secretion (1.5 to 37-fold) in the conditioned medium of HPMC cultures primed with ovarian cancer ascites (Fig. 2d). Of note, OVC439 and OVC509 ascites, which had a modest stimulation of MUC16 expression by immunoblot (Fig. 2a), failed to induce a significant increase of MUC16 release relative to the control. Importantly, peritoneal fluids from women with benign conditions (*n* = 2) failed to stimulate the secretion of MUC16 in HPMCs suggesting that the ascites factors responsible for the stimulation of MUC16 release may be absent or present at a lower concentration in benign peritoneal fluids. Heat-inactivation of ovarian cancer ascites almost completely abrogated their ability to stimulate MUC16 secretion suggesting that the MUC16-stimulating factor(s) in ascites are most likely proteins (Fig. 2e). Next, we determined MUC16 mRNA levels in response to benign fluid OV401 and malignant ascites OVC346 and OVC690. Fluids were added at a concentration of 10% (*v*/v) and cells were treated for 4 or 8 h. MUC16 mRNA expression was not stimulated by the presence of HGSOC ascites (Fig. 2f). Indeed, MUC16 mRNA levels were not significantly different in ascites-treated HPMCs versus cells incubated with serum (control) or benign fluid OV401. These data suggest that most HGSOC ascites consistently stimulate the protein expression and release of MUC16 in HPMC cultures without affecting MUC16 mRNA levels. To examine the clinical relevance of these in vitro findings, we stained omental biopsies from patients with non-inflammatory conditions and omental biopsies from patients with HGSOC for MUC16 expression. As shown in Fig. 2g, MUC16 expression was only detectable on the mesothelium lining of omental biopsies from patients with HGSOC.

### Ascites stimulate MUC16 secretion in HPMCs in a dose-dependent manner

**Fig. 3:**
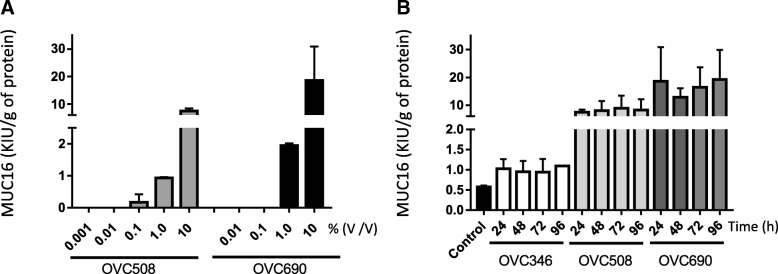
Dose-dependent release of MUC16 in HPMCs treated with ascites (0,001–10%). **a** Meso7 cells were incubated with increasing concentrations of OVC508 or OVC690 ascites overnight, cells were rinsed 3 times and MUC16 shedding was determined in the conditioned medium 24 h later. **b** Meso7 cells were incubated overnight with ascites (10% *v*/v), rinsed 3 times and MUC16 shedding was determined after 24 h and up to 96 h post treatment. Levels were standardized according to the total protein concentration. Results are from two independent experiments performed in duplicates

Next, we determined the dose responsiveness of MUC16 secretion to ovarian cancer ascites (Fig. 3a). Ovarian cancer ascites OVC508 and OVC690 were selected because of their strong MUC16-stimulating effect. Ascites were added at concentrations ranging from 0.01 to 10% *v*/v to HPMC cultures and the release of MUC16 in the supernatant was measured. In these experiments, OVC508 and OVC690 ascites concentration as low as 1% resulted in a significant stimulation of MUC16 release. At 10% concentration, each ascites induced a very robust stimulation of MUC16 release (> 18 and > 44-fold respectively). Maximal stimulation of MUC16 release was reached within 24 h following exposure to ascites. Longer exposure to ascites did not significantly increased MUC16 release in the supernatant of HPMC cultures (Fig. 3b).

### Akt activation but not NF-κB is involved in ascites-induced MUC16 expression

**Fig. 4:**
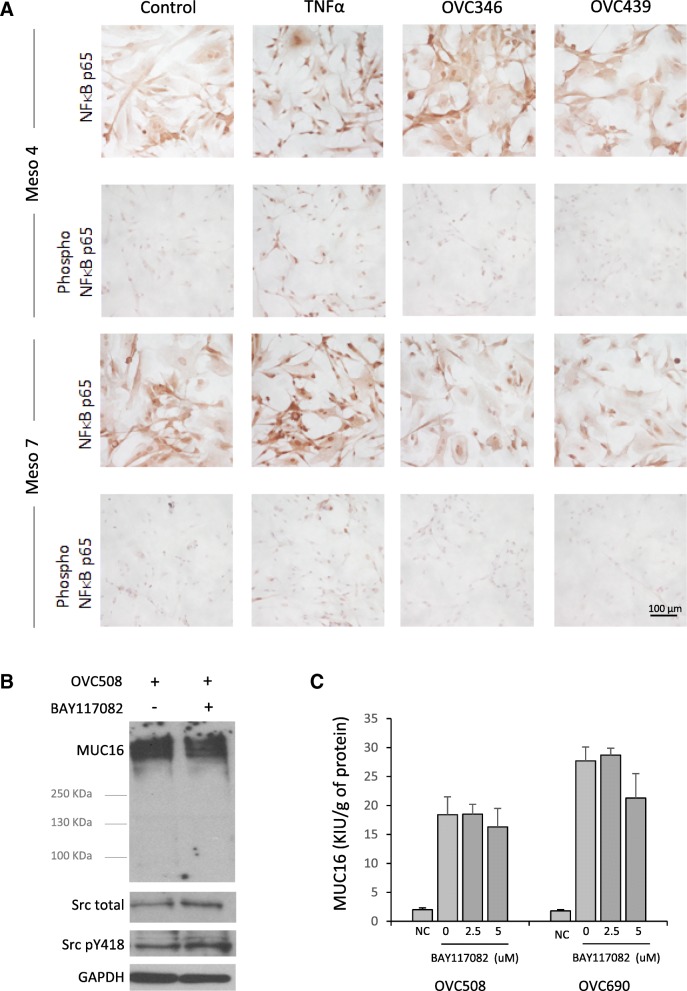
NF-κB activation in HPMCs. **a** Total and phosphorylated NF-κB p65 expression and localization were determined in two HPMCs samples (Meso 4 and Meso 7 cells) using anti-NF-κB p65 specific antibodies. HPMCs were incubated with either FBS (10%), TNF-α (20 ng/ml), OVC346 (10%) or OVC439 (10%) for 20 min. **b** Meso7 cells were incubated with OVC508 ascites (10% v/v) in the presence or absence of NFκB inhibitor BAY117082 (5 μM) for 24 h. Cells were then washed three times with PBS and incubated with standard medium for 24 h. Lysates were then obtained and immunoblotted for MUC16, c-Src and phosphorylated (Y418) c-Src. GAPDH was used as a loading control. **c** Meso 7 cells were pretreated or not with BAY117082 (2 or 5 μM) for 1 h prior to the addition of ascites OVC508 or OVC690 (10%) to the medium. Cells were incubated overnight. The next day, cells were washed 3 times and incubated 24 h with standard medium. The medium was removed and MUC16 concentration were measured in the condition medium. Results are from two independent experiments performed in duplicates

MUC16 expression has been shown to be NF-κB-dependent in some cancer cell lines [26, 38]. Because we had evidence that NF-κB**-**mediated transcription is stimulated by ascites in ovarian cancer cell lines (Lane, unpublished data), and because NF-κB is a downstream effector of several cytokines, we assessed the role of NF-κB in ascites-induced stimulation of MUC16 expression and release. To this end, we assessed, by immunohistochemistry, the nuclear localization of NF-κB family member RelA (p65), which is phosphorylated and accumulates in the nucleus when the NF-κB pathway is activated. As shown in Fig. 4a, TNFα, a well-known NF-κB activator, induced the phosphorylation and accumulation of p65 in the nucleus of HPMC cultures. In contrast, incubation of HPMC cultures with ovarian cancer ascites displayed p65 nuclear accumulation that were similar to the control suggesting that ascites do not stimulate NF-κB activity in HPMCs. As c-Src is an upstream activator of NF-κB pathway, we assessed whether ascites could activate c-Src. As shown in Fig. 4b, ascites failed to induce c-Src which is consistent with data from Fig. 4a. In line with these data, NF-κB inhibitor BAY117082 had no effect on ascites-induced MUC16 protein expression (Fig. 4b) and release (Fig. 4c). Collectively, these data demonstrate that ascites-induced MUC16 expression and release is independent of NF-κB activation.

**Fig. 5:**
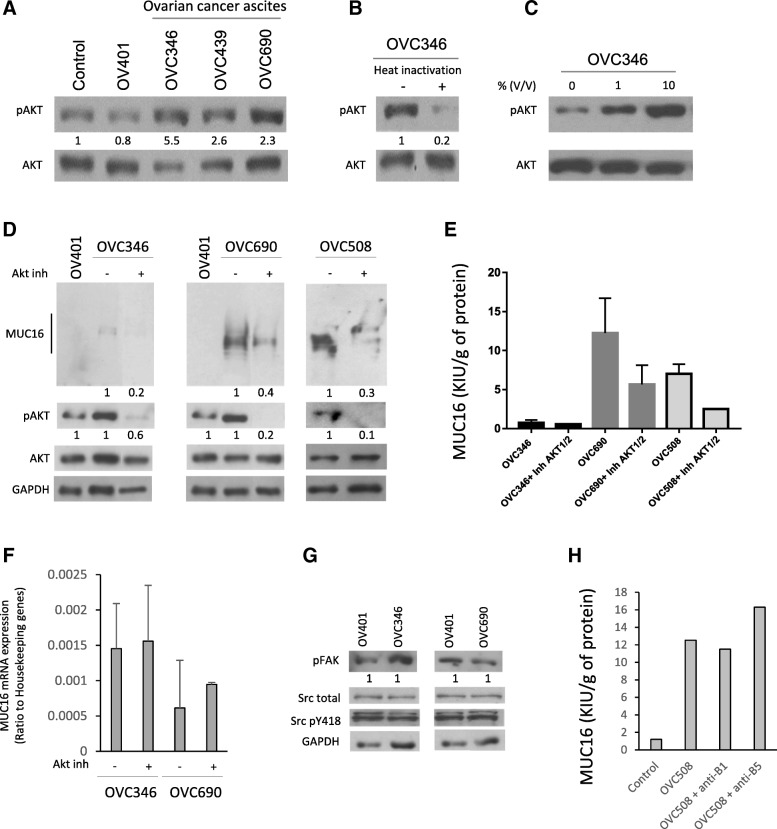
Ovarian cancer ascites stimulate MUC16 expression and release through Akt. **a** Meso 7 cells were incubated with FBS 10% (control), OV401 10% or 3 different ovarian cancer ascites at 10%. The expression of Akt and phosphorylated Akt were determined by immunoblot using anti-Akt and anti-phospho-Akt antibodies. Representative images from two independent experiments. Densitometric quantification of phosphorylated Akt normalized to total Akt is represented between the two blots. **b** OVC346 ascites was heat-inactivated by heating at 100 °C for 10 min followed by centrifugation at 13,000 rpm for 15 min. Meso7 cells were incubated with OVC346 (10%) or heat-inactivated OVC346 ascites (10%). Akt and phosphorylated Akt expression were assessed by immunoblot after 24 h. Expression of phosphorylated Akt was normalized to total Akt and densitometric quantification is shown between the blots. Representative images from two independent experiments. **c** Meso 7 cells were incubated with increasing concentrations of OVC346 ascites (0 to 10% v/v) and Akt and phosphorylated Akt expression were determined by immunoblot. **d** Meso7 cells were incubated with OV401 (10%) or ovarian cancer ascites (10%) in the presence or absence of Akt1/2 inhibitor (5 μM). MUC16, Akt and phosphorylated Akt expression were determined by immunoblot. Representative images from two independent experiments. Expression of phosphorylated Akt was normalized to total Akt and MUC16 expression was normalized to housekeeping gene GAPDH. **e** MUC16 release was measured in supernatants of Meso7 cells incubated with three different ovarian cancer ascites in the presence or absence of Akt1/2 inhibitor (5 μM). Bars represent standard deviation from two independent experiments conducted in duplicates. **f** Meso7 cells were incubated with OVC346 or OVC690 (10%) in the presence or absence of Akt1/2 inhibitor. MUC16 mRNA levels were quantified by ddPCR. MUC16 concentration data from ddPCR experiments are expressed as ratio against reference genes for each sample. Results are from two independent experiments conducted in duplicates. **g** Meso7 cells were incubated with benign fluid OV401 or ovarian cancer ascites OVC346 or OVC690 (10%). Expression of phosphorylated FAK, phosphorylated and total c-Src was determined by immunoblot. Representative images from two independent experiments. Densitometric quantification of phosphorylated FAK normalized to GAPDH is shown between the blots. **h** Cells were incubated with β1 or β5 integrin-blocking antibodies (5 μg/ml) for 30 min before adding 10% OVC508 ascites overnight. Cells were then washed 3 times and fresh FBS and hormone-free medium was added. MUC16 release was then measured in supernatants after 24 h. Data are from a single experiment

Ovarian cancer ascites induces Akt activation in ovarian cancer cells [39, 40]. Hence, we speculated that ascites-mediated increased MUC16 expression in HPMC monolayer cultures might be dependent on Akt activation. To test this hypothesis, we assessed Akt activation in HPMCs incubated with ovarian cancer ascites (*n* = 3). As shown in Fig. 5a, ascites induced Akt activation as compared to benign fluid (OV401) or control by at least a 2-fold. Heat inactivation drastically reduced OVC346 ascites-induced Akt activation (Fig. 5b). In line with data from Fig. 3a, we observed a dose-dependent phosphorylation of Akt with increasing concentration of ascites (Fig. 5c) indicating that ovarian cancer ascites stimulate Akt activation in HPMCs. To confirm the hypothesis that ascites-stimulated MUC16 expression and release is mediated through an Akt-dependent pathway, we examined the ability of the Akt1/2 specific inhibitor, 1 L-6-hydroxymethyl-*chiro*-inositol 2(R)-2-*O*-methyl-3-*O*-octadecylcarbonate, to inhibit ascites-enhanced MUC16 expression and ectodomain release. Akt inhibition decreased ascites-induced MUC16 protein expression by at least 2-fold (Fig. 5e) and significantly inhibited (~ 30%) MUC16 shedding, suggesting that ascites-mediated MUC16 expression and shedding involve, at least partially, an Akt-dependent signaling pathway. As expected, Akt inhibition had no effect on the level of MUC16 mRNA expression (Fig. 5f).

Because we have previously shown that ascites-induced Akt activation in ovarian cancer cells is mediated by αvβ5 integrin and focal adhesion kinase (FAK) activation [40], we determined whether ascites that strongly promote MUC16 expression and secretion could induce FAK activation in HPMCs. As shown in Fig. 5g, OVC346 and OVC690 ascites failed to enhance FAK phosphorylation and its downstream target c-Src in HPMCs. Consistent with these findings, the addition of blocking antibodies anti-β1 and anti-β5 had no effect on OVC508 ascites-induced MUC16 release (Fig. 5h) thus confirming that ascites-induced Akt activation in HMPCs is not mediated through an integrin/FAK pathway.

### Ascites factor(s) associated with MUC16 release

The lack of stimulation of MUC16 release by benign peritoneal fluids (Fig. 2d) suggest that factors differentially expressed between ovarian cancer ascites and benign peritoneal fluids could be responsible for ascites-induced stimulation of MUC16 expression and release. To identify inflammation-related factors in ascites that might be responsible for stimulating MUC16 release, we performed a cytokine array on ascites samples. Among the 120 cytokines tested, the top ten differentially expressed between MUC16-stimulating and none-stimulating fluids were IL-6, CXCL1, CCL20, hepatocyte growth factor (HGF), IL-16, IL-1R4, CCL7, CCL8, CCL16 and IL-10 (Table 2). None-stimulating peritoneal fluids were defined as those with less then two-fold MUC16 release increased in conditioned medium relative to the control, which included OV401, OV437, OVC439 and OVC509 fluids. Incubation of HPMC monolayer cultures with recombinant IL-6, HGF, CCL7, CCL8, CCL16, CCL20, CXCL1 and IL-10 at biologically relevant concentrations failed to stimulate MUC16 protein expression or its release in the supernatant of HPMC cultures (Table 3). Furthermore, incubation of HPMCs with recombinant CCL18 and Leptin, two molecules previously shown to alter HPMC signaling [41, 42], had no effect on MUC16 expression and release (Table 3). In line with these data, antibody-mediated inhibition of IL-6 and HGF did not altered MUC16 release (data not shown).

**Table 2:** Top ten factors differentially expressed between MUC16-stimulating and non-stimulating ascites (*P* <  0.05)

Factors	MUC16-nonstimulating Mean^a^ (± SEM) (*n* = 5)	MUC16-stimulating Mean (± SEM) (*n* = 2)	Fold increased	*P* value
IL-6	2545 (± 399)	24,357 (± 802)	9.6	< 0.0001
CXCL1	3736 (± 247)	26,265 (± 4506)	7.0	0.0002
CCL20	656 (± 31)	4525 (± 666)	6.9	< 0.0001
HGF	2321 (± 450)	10,561 (± 1069)	4.6	< 0.0001
IL-16	788 (± 71)	3344 (± 675)	4.2	0.0021
IL-1 R4	948 (± 59)	3957 (± 850)	4.2	0.0033
CCL7	571 (± 23)	2276 (± 322)	4.0	0.0001
CCL8	548 (± 23)	1901 (± 276)	3.5	0.0002
CCL16	1152 (± 166)	3886 (± 627)	3.4	0.0009
IL-10	2328 (± 713)	7701 (± 1144)	3.3	0.0014

^a^Means are expressed in relative fluorescence units

**Table 3:** Effect of selected soluble factors on MUC16 release in HPMCs

Factors	Concentrations	Stimulation of MUC16 release
IL-6	2 ng/ml	No
IL-10	1 ng/ml	No
TNFα	25 ng/ml	No
Leptin	500 pg/ml	No
HGF	1 ng/ml	No
CCL7	10 ng/ml	No
CCL8	10 ng/ml	No
CCL16	10 ng/ml	No
CCL20	10 ng/ml	No
CXCL1	10 ng/ml	No

## Discussion

Despite the fact that the measurement of MUC16 extracellular domain (CA125) in the sera has been the mainstay of HGSOC assessment and management since the early 1980’s, there are considerable gaps in our knowledge regarding the regulation of MUC16 expression and release in the context of ovarian cancer. Given the immunoprotective role of MUC16 ectodomain, identifying how the tumor environment alters MUC16 expression and secretion in cancer-associated stromal cells may provide a new way to block immune evasion by tumors. In the present study, we demonstrate for the first time that HGSOC ascites, a highly inflammatory tumor environment, markedly up-regulates the expression of MUC16 glycoprotein in primary HPMCs and stimulate its release from the cell surface. The regulation of ascites-mediated upregulation of MUC16 expression occurs at the post-transcriptional level. Furthermore, we show that ascites-induced stimulation of MUC16 expression is largely mediated by the activation of Akt pathway in HPMCs. Although we identified a number of cytokines, chemokines and growth factors preferentially expressed in MUC16-stimulating ascites versus those without stimulating effects, we were unable to demonstrate that any of these factors were responsible for ascites-induced MUC16 expression in HPMCs.

MUC16 may be expressed by various tumor cell types and by some normal epithelial cells. Expression of MUC16 has been reported in ocular surface epithelial cells lines [29, 43, 44] and tracheal surface epithelium [45]. Although cytokines may be involved in MUC16 shedding by ocular cells, the regulation of MUC16 shedding by cytokines appears to be complex. For example, whereas IL-1α, IL-1β and TNFα failed to increase MUC16 mRNA levels in human corneal cells, treatment with these cytokines was nonetheless associated with increased MUC16 shedding [28]. In contrast, treatment of human conjunctival cells with IL-1α, IL-1β and TNFα reduced MUC16 shedding despite increasing MUC16 mRNA levels. The data suggest that cytokines may produce several patterns of MUC16 regulation in normal epithelial cells in which the amount of MUC16 in cells and the amount of MUC16 shedding do not always correlate. Therefore, depending on the cellular context, MUC16 shedding may be increased without changes in mucin mRNA levels. Our data show that there is good correlation between MUC16 protein levels and ectodomain release suggesting that MUC16 is upregulated suggesting that ascites regulates MUC16 expression at a post-transcriptional level.

The stimulation of MUC16 release by cytokines in various cancer cell lines, including, endometrial, breast and ovarian tumor cells have also been variable. TNFα and IFNγ, the two most studied cytokines in that context, have been shown to enhance MUC16 shedding in some but not all cancer cell lines suggesting also a complex regulation of MUC16 expression that is dependent on the cellular context. NF-κB has been involved in cytokine-mediated stimulation of MUC16 release in cancer cells [26]. In contrast to this finding, we show here that TNFα does not affect MUC16 expression and release in HPMCs. Furthermore, we demonstrated that although NF-κB may be activated by ascites in OC cells, this pathway is not involved in ascites-mediated MUC16 stimulation in HPMCs as demonstrated by the lack of ascites-mediated NF-κB p65 phosphorylation and the lack of effect of NF-κB specific inhibitor BAY117082 on MUC16 expression. Based on these observations, it appears that although both tumor and mesothelial cells may produce MUC16 under inflammatory conditions; the mechanisms regulating MUC16 expression differ between these cells.

In this study, we show that MUC16 expression is, at least partly, regulated by Akt activation as demonstrated by the observation that ascites induce Akt activation in a dose-dependent manner and the fact that a specific Akt inhibitor strongly reduced MUC16 expression and release. The mechanism by which Akt stimulate MUC16 expression and release remains unclear. Serum CA125 correlates with progression and regression of the disease and this formed the basis for monitoring CA125 serum levels for patient follow-up [7, 8]. However, the fact that mesothelial cells are a major source of secreted CA125 in a pro-inflammatory surrounding environment such as ascites would suggest that rising and falling CA125 levels merely reflect the degree of inflammation in a patient rather than the tumor burden itself.

## Conclusions

In summary, our data reveal that peritoneal mesothelial cells may be an important source of MUC16 when exposed to HGSOC ascites, but the regulation of MUC16 expression appears to be complex. In addition, although ascites regulate MUC16 expression at a post-transcriptional level through an Akt-dependent pathway, the ascites factors involved in MUC16 expression upregulation remains to be characterized.

## Abbreviations

CA125: Cancer antigen 125
CCL: Chemokine (C-C motif) ligand
CTD: C-terminal domain
CTRNet: Canadian Tumor Repository Network
CXCL: Chemokine (C-X-C motif) ligand
ddPCR: droplet Digital PCR
FAK: Focal adhesion kinase
FIGO: International Federation of Gynecology and Obstetrics
HGF: Hepatocyte growth factor
HGSOC: High grade serous ovarian cancer
HPMCs: Human peritoneal mesothelial cells
IFN: Interferon
IL: Interleukin
MMP: Metalloprotease
NE: Neutrophil elastase
NF-κB: Nuclear factor κB
NK: Natural killer
OC: Ovarian cancer
TNF: Tumor necrosis factor
